# Administration of human macrophage colony-stimulating factor-mFc fusion protein increases macrophage phagocytic capacity and stimulates liver regeneration in mice

**DOI:** 10.1186/s12950-026-00507-8

**Published:** 2026-06-25

**Authors:** Philip J. Starkey Lewis, Jennifer A. Cartwright, Lara Campana, Birgit U. Baumgarten, Hilmar Ebersbach, Marie-Gabrielle Ludwig, Gregory Marszalek, Benjamin Stutchfield, Rhona Aird, Tak-Yung Man, Eoghan O’Duibhir, Klaus Seuwen, Stuart J. Forbes

**Affiliations:** 1 Centre for Regenerative Medicine, Institute for Regeneration and Repair, University of Edinburgh, Edinburgh, UK; 2 Centre for Inflammation Research, Institute for Regeneration and Repair, University of Edinburgh, Edinburgh, UK; 3 The Royal (Dick) School of Veterinary Studies and the Roslin Institute, University of Edinburgh, Edinburgh, UK; 4 Novartis Institutes for Biomedical Research, Basel, Switzerland

**Keywords:** Macrophages, Phagocytosis, Colony-stimulating factor-1, Carbon tetrachloride, Interleukin-10

## Abstract

**Background:**

Acute and chronic liver diseases are rising contributors of the worldwide healthcare burden. Inflammation plays a key role in progression of both acute and chronic disease, and effective treatments are limited. Macrophages, a diverse population of professional phagocytes, are required for the timely resolution of tissue injury and are an emerging target to treat acute and chronic liver disease. Colony stimulating factor 1 (CSF1), promotes differentiation and survival of macrophages, which are essential in epithelial regeneration, but the CSF1 protein is rapidly eliminated in vivo. We therefore fused human CSF1 genetically to a modified mouse IgG2a Fc region and transiently expressed this in HEK293-6E cells. The protein was purified from culture supernatant to obtain > 97% monomeric purity, suitable for in vivo testing.

**Results:**

The nascent hCSF1-Fc fusion proteins were evaluated in vitro and in murine models of health and liver disease. hCSF1-mFc is pharmacologically active in healthy mice, resulting in increases in liver and spleen mass closely reflecting changes in tissue macrophage content. In an acetaminophen-induced acute liver injury model, hCSF1-mFc treatment increased hepatic macrophage accumulation at necrotic sites, reduced serum ALT activity, and serum IL-1β levels, and restored white blood cell counts within 72 h. Furthermore, hCSF1-mFc treatment also increased hepatic macrophage numbers in a carbon tetrachloride (CCl_4_)-induced liver fibrosis model corresponding with a striking 3.5-fold increased uptake of a soluble phagocytic label, indicating improved hepatic clearance capacity. hCSF1-mFc was also tested in a model of acute on chronic liver injury, which included a bolus delivery of proinflammatory *E. coli*-coated bioparticles to pre-existing fibrosis. Here, hCSF1-mFc treatment restored body weight, induced hepatomegaly, and reduced several plasma pro-inflammatory cytokines, suggesting reduction of systemic inflammation. Finally, immunofluorescence staining showed hCSF1-mFc treatment induced significant hepatocyte proliferation in healthy and all liver disease models.

**Conclusion:**

This report demonstrates that hCSF1-mFc treatment increases liver macrophage populations, improves phagocytic capacity, and stimulates hepatocyte proliferation in experimental liver disease models. hCSF1-based fusion protein therapy might represent a novel immune-based strategy to treat acute and chronic liver disease in the clinic.

**Clinical trial number:**

Not applicable.

**Supplementary Information:**

The online version contains supplementary material available at https://doi.org/10.1186/s12950-026-00507-8.

## Background

Deaths attributed to liver disease continue to rise and represent an increasing significant worldwide healthcare burden [1]. Chronic liver diseases, including cirrhosis of the liver, account for the most cases of liver transplantations performed in the United States and United Kingdom, with an additional number arising to treat acute liver failure (ALF) [2, 3]. Whilst orthotopic liver transplantation is a life-saving treatment for end stage liver disease, it is constrained by a shortage of suitable donor organs, a risk of post-operative complications, and commits patients to lifelong immunosuppression [4]. Therefore, new therapeutic strategies are required to treat end-stage liver disease.

Tissue resident macrophages play an important role in tissue homeostasis where they serve as professional phagocytes to clear noxious agents and also possess trophic functionality to regulate and repair the local tissue microenvironment [5]. Liver resident macrophages (Kupffer cells, [KCs]) are highly phagocytic and provide a critical immunological barrier by efficiently clearing enteric bacteria and their components (e.g. lipopolysaccharides) from the portal blood supply. Recent evidence shows that KCs becomes compromised following acute toxic liver injury (ALI) resulting in a diminished hepatic barrier function [6–8]. Increased bacterial translocation resulting from impaired gut wall integrity predisposes liver disease patients to systemic infections, which are major determinants of mortality in both acute and chronic disease settings [9–13]. Since macrophages play distinct roles in the initiation and resolution of tissue injury and inflammation, and have the propensity to modulate the liver microenvironment, they have been described as potential therapeutic targets for liver disease [14]. Recently, experimental work in pre-clinical models supported by the development of GMP-compliant protocols to manufacture human macrophages have led to clinical trials using macrophages as a cell therapy for liver disease [15–19]. Macrophage colony-stimulating factor (CSF1) is a potent cytokine that controls tissue-resident macrophage populations by stimulating the macrophage CSF1 receptor (CSF1R), together with IL-34, to promote macrophage proliferation and survival [20, 21]. CSF1R-dependent macrophages have been shown to be essential in epithelial regeneration [22]. Previously, in liver disease models, evaluation of CSF1 was constrained by the rapid elimination of the protein in vivo [23]. However, fusion proteins that conjugated animal CSF1 proteins to the Fc region of immunoglobulins were recently described, which conferred a vastly improved pharmacokinetic profile in pre-clinical species [24, 25]. Porcine CSF1-Fc has been shown to induce hepatosplenic macrophage expansion and hepatocyte proliferation in healthy mice [24]. Reversible expansion of tissue macrophages has also been documented in other tissues in response to CSF1-Fc fusion protein with impacts on metabolism [26]. Previous work demonstrated that both porcine CSF1-Fc and human CSF1-Fc treatment increased tissue macrophage number in a mouse thioacetamide-induced liver fibrosis model and increased hepatic proliferation and liver regeneration following injury [27]. These studies demonstrate that CSF1-Fc fusion proteins have potent activity in chronic experimental disease models, but highlight a requirement to better understand the mechanistic action of human CSF1-Fc fusion protein treatment, particularly in acute liver conditions, to broaden its potential clinical utility.

Herein, novel CSF1-Fc fusion proteins were synthesised by conjugating human CSF1 (hCSF1; amino acids 36–190) to either Fc regions of immunoglobulins for either mouse (Fc2a) or human Fc (Fc1P). We tested these nascent fusion proteins have in healthy mice, and in murine models of acute and chronic liver injury to evaluate safety and efficacy. We report that hCSF1-mFc shows potent bioactivity in mice evidenced by a rapid and striking increase in hepatic macrophage numbers within 72 h and led to increased hepatic phagocytic capacity. Furthermore, hCSF1-mFc treatment reduced proinflammatory cytokine concentrations, reduced circulating transaminase levels, and stimulated hepatocyte proliferation in acute and chronic liver injury models. Therefore, we demonstrate that human CSF1-Fc treatment boosts hepatic macrophage populations to improve phagocytic clearance capacity and stimulate parenchymal regeneration. These findings provide evidence that hCSF1-based fusion proteins might have therapeutic value and clinical utility in the context of liver disease.

## Materials and methods

### hCSF1 fusion protein production

Human CSF-1 (uniprot P09603; aa36-190) was genetically fused to the Fc part of mouse IgG2a (mIgG2a-Fc) including the hinge region (Genebank CAA49868.1; aa215-447) via a linker. The linker consists of a PreScission protease cleavage site for potential removal of mIgG2a-Fc and short linkers upstream and downstream of the cleavage site (GSLEVLFQGPGSS). To reduce binding of the fusion construct to high and low affinity mouse Fc gamma receptors, two mutations were introduced in the CH2 domain of mIgG2a-Fc, at positions 234 and 235 (L234A/L235A) [28]. The complete construct (hCSF-1_mIgG2a-Fc) was cloned into a mammalian expression vector, including a signal peptide/leader of CD33. Large scale transient expression in HEK293-6E cells was initiated by polyethylenimine plasmid transfection and subsequent cultivation of transfected cells for approximately 5 days including feeding. Cell viability at 75% triggered harvest and filtration, resulting in a titre of approximately 60 mg/L culture volume. Supernatant was loaded overnight onto a 300 mL self-packed MabSelect Sure column. After a baseline wash, the protein was eluted with 50 mM citrate/140 mM NaCl (pH 2.7) and the eluate pH was immediately adjusted to 7.4 and subsequently sterilely filtered. Analytical Size-Exclusion-Chromatography (SEC) showed a monomeric fraction of 60%, which triggered a polishing step by preparative SEC. A third of the MabSelect Sure column eluate was concentrated on 30 kDa cut-off spin filters to 5 mL. Concentrate was applied to a 26/600 Superdex 200 column using PBS (pH 7.4) as running buffer, at a flow rate of 1.5 mL/min, and 4 mL fractions were collected. Analytical SEC was performed and fractions with the highest amount of monomeric content were pooled, resulting in final protein purity of > 97%. Protein identity was confirmed by mass spectroscopy.

### Mice

All mice (C57Bl/6J OlaHsd males, aged 6–10 weeks old) were sourced from Envigo (Indianapolis, IN) and group-housed in open top cages with free access to chow and water. Experimental mice were humanely euthanized, before complete exsanguination to collect trunk blood *via* cardiac puncture. All animal experiments were undertaken in accordance with criteria outlined in the procedure project license (70/7847 and P231C5F81) granted by the Home Office (United Kingdom) under the Animals (Scientific Procedures) Act 1986 and approved by the University of Edinburgh Animal Welfare Ethical Review Body.

### Experimental APAP-ALI

Mice were fasted for a minimum of 12 h before receiving APAP (350 mg/kg, i.p.; Sigma-Aldrich, St. Louis, MO) dissolved in warm saline (PanReac Applichem, Germany) to induce ALI. Chow was returned to cages 20 min post-APAP administration. APAP-ALI mice were randomized to receive hCSF1-mFc (1.0 mg/kg, s.c.) or vehicle alone (Dulbecco’s Phosphate-Buffered Saline [PBS]; Gibco, Carlsbad, CA) at 12, 36, and 60 h post-APAP (*n* > 6 per treatment group). Mice received thermal support post-APAP by accommodating cages in a heated cabinet (28 °C) to limit hypothermia. All mice were labelled with BrdU (1 mg, i.p.) one hour before cull at 72 h post-APAP.

### Carbon tetrachloride-induced experimental liver fibrosis

Liver fibrosis was induced in mice by iterative twice-weekly carbon tetrachloride (CCl_4,_ 0.6 µL/g; Sigma-Aldrich; diluted 1:4 in sterile olive oil) administration for seven weeks. Fibrotic mice were randomized to receive 3 doses of hCSF1-mFc (0.1–1.0 mg/kg, s.c.), or vehicle alone, (PBS, *n* = 4–7 per treatment group) over 72 h. All mice were labelled with BrdU (1 mg, i.p.) one hour before cull at 72 h post-CCl_4_-induced fibrosis. In some experiments, mice received PKH26-PCL, (PKH26 Red Fluorescent Cell Linker Kit for Phagocytic Cell Labelling) (100 µL, 0.1 mM, i.v.; Sigma-Aldrich) 24 h prior to cull to label cells performing phagocytosis as described [29].

### Liver fibrosis with acute insult

To recapitulate an acute on chronic liver insult, the the seven-week CCl_4_ fibrosis protocol was modified in some studies to include a single administration of *E. coli*-coated fluorescent pHrodo^®^ bioparticles (100 µg, i.v.; Thermo Fisher, Waltham, MA), alongside the final CCl_4_ or control injection.

### Serum and plasma biochemistry

Serum was collected by allowing whole blood to clot overnight (4 °C) before centrifugation (14,000 g, 5 min, 4 °C). Plasma was generated by collecting whole blood in EDTA blood tubes and collected after immediate centrifugation (6,000 g, 10 min, 4 °C). Serum and plasma were assayed for alanine aminotransferase (ALT), aspartate aminotransferase (AST), and alkaline phosphatase (ALP) activities alongside albumin and total bilirubin determination using commercial kits (Alpha Laboratories Ltd, United Kingdom; Randox Laboratories, United Kingdm) adapted for use on a Cobas Fara centrifugal analyser (Roche Diagnostics, Basel, Switzerland) as previously described [30, 31].

### Cytokine quantification

Cytokine panels were quantified in serum, plasma, and liver homogenates using commercial kits (Meso Scale Diagnostics, Rockville, MD) on a MESO Quickplex SQ 120 analyser (Meso Scale Diagnostics) following the manufacturers instructions, as described previously [17].

### Immunodetection, imaging, and other assays

Please refer to the supporting materials for further materials and methods.

### Statistical analysis

Statistical analysis was performed in Prism 8.2 (GraphPad Software, San Diego, CA). In charts, scatter plots display each experimental unit, unless otherwise stated. Shapiro-Wilk test determined normality in datasets. When comparing two datasets, a Student’s *t*-test or a Mann-Whitney U test was performed on parametric or non-parametric datasets, respectively. When comparing three or more datasets, a one-way ANOVA was performed on parametric datasets with Dunnett’s post-hoc testing, otherwise a Kruskall-Wallis test was performed on non-parametric datasets. *P* < 0.05 was considered statistically significant.

## Results

### hCSF1-mFc administration induces macrophage accumulation in healthy mouse liver

**Fig. 1:**
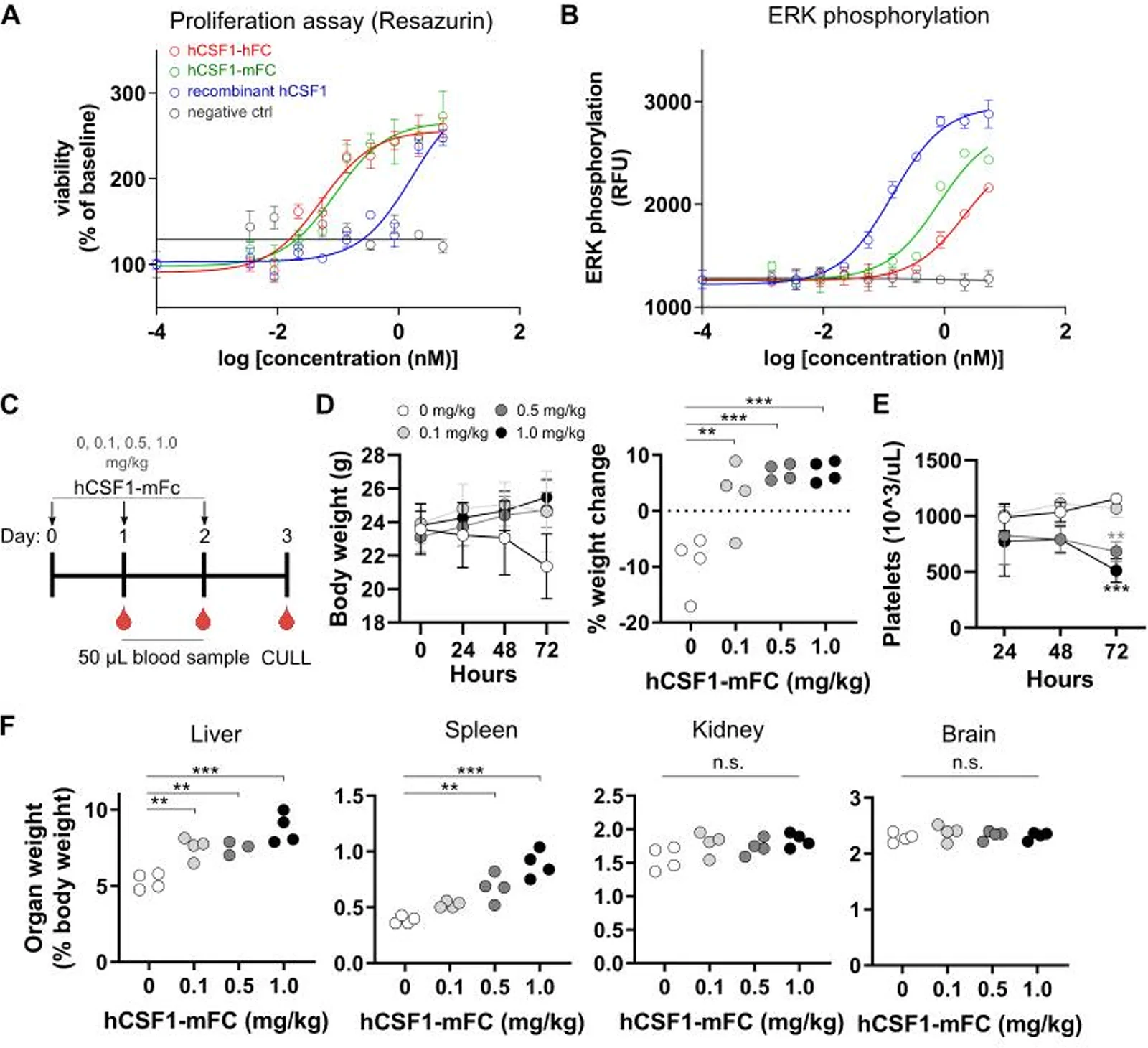
hCSF fusion proteins show bioactivity in vitro and in vivo. (**A**) Human monocytes were isolated from healthy volunteer buffy coats and treated in culture with hCSF1-mFc (green) (EC50 0.052, CI, R^2^ 0.96), hCSF1-hFc (red) (EC50 0.09, R^2^ 0.96), human recombinant CSF1 (blue) (EC50 1.689, R^2^ 0.9), or a negative control fusion protein (anti-chi-lysozyme-MOR3207-hIgG1-LALA; grey) for 72 h. Data standardised to lowest treatment concentration. (**B**) ERK phosphorylation quantified in human monocytes treated with hCSF1-mFc proteins for 5 min. Non-linear regression curves are plotted for each treatment group; hCSF1-mFc (EC50 2.25, R^2^ 0.99, hCSF1-hFc (EC50 0.81, R^2^ 0.95), human recombinant CSF1 (EC50 0.14, R^2^ 0.99). (**C**) Study design for in vivo experiment: Healthy C57BL/6J males were administered hCSF1-mFc (0–1.0 mg/kg, s.c.) once-daily for three consecutive days. Mice had blood microsampled daily following treatment start and tissues/biofluids were analysed 24 h after the final dose. (**D**) Average daily body weight of littermates are shown per treatment group (left panel) with individual percentage weight changes (right panel). (**E**) Average daily blood platelet counts per treatment group (Geisser-Greenhouse correction, and Dunnett’s post-hoc test). (**F**). Final organ weights are shown as a percentage of body weight for individual mice in groups for indicated organs. Circles represent individual mice in charts. Clear circles, 0.0 mg/kg (vehicle alone); pale grey circles, 0.1 mg/kg; dark grey circles, 0.5 mg/kg; black circles, 1.0 mg/kg. Shapiro-Walk normality test and ANOVA performed, Dunnett’s post-hoc test. Data expressed as mean ± SD, unless otherwise stated. n.s., not significant; ** *P* < 0.01; *** *P* < 0.001. Abbreviation: ERK, extracellular signal-regulated kinase

Firstly, hCSF1 fusion proteins were synthesised by combining amino acids 36–190 of the human protein to either mouse (mFc2a) or human (hFc1P) Fc fragments. To confirm that the nascent fusion proteins were biologically active, hCSF1-mFc and hCSF1-hFc were tested in vitro alongside commercially sourced recombinant hCSF1 and vehicle alone (negative control) in cultured human monocytes freshly isolated from healthy volunteers. Both hCSF1 constructs increased resazurin turnover at lower concentrations than recombinant hCSF1 alone, demonstrated by EC50s (Fig. 1A). Reasurin floursence is reduced by metabolically active cells and signal intensity is proportional to viable cell number. Increased monocyte proliferation suggests potent bioactivity of hCSF1 constructs *in vitro.*

**Fig. 2:**
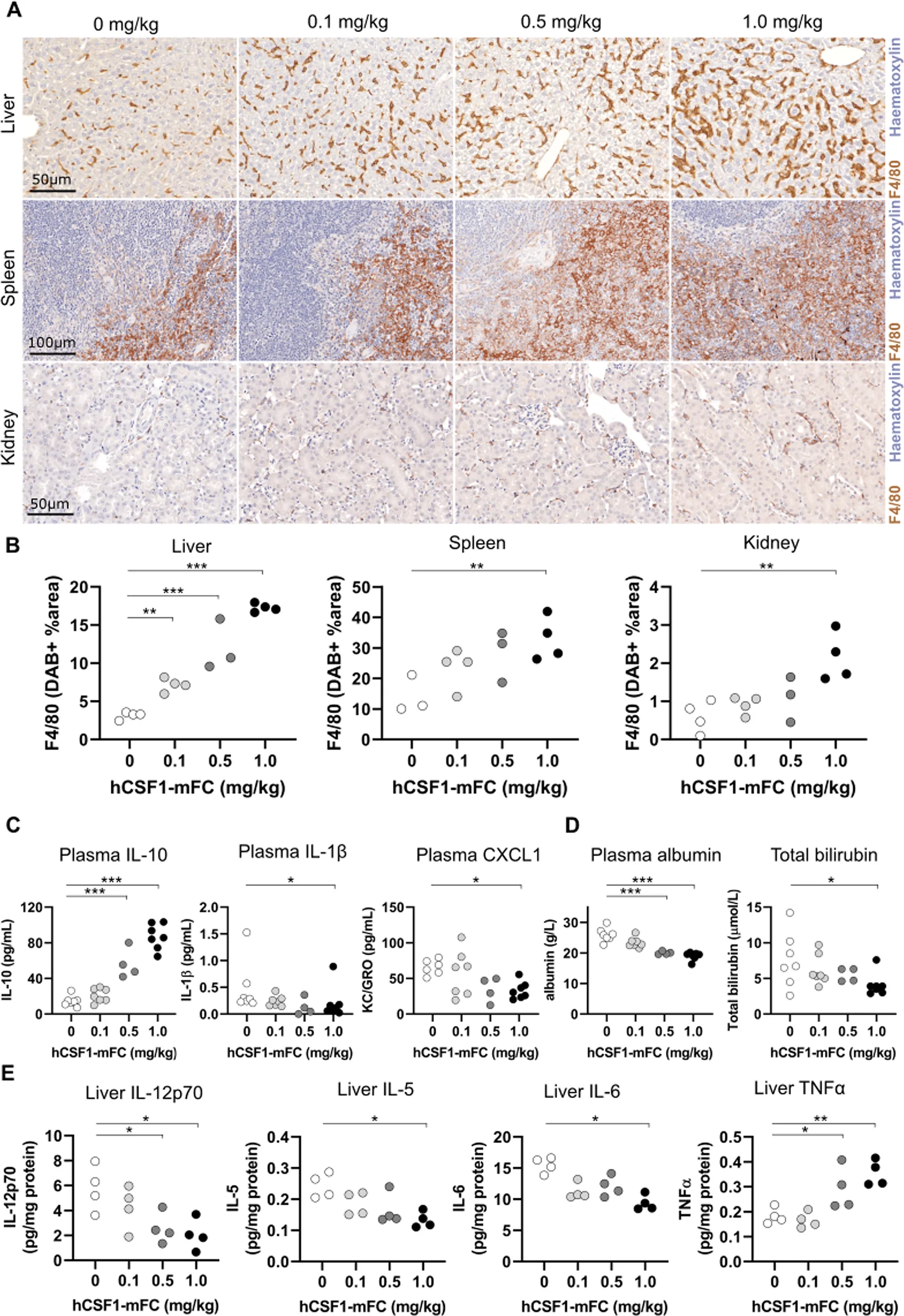
Iterative hCSF-mFc treatment increases hepatic macrophage numbers and influences circulating and hepatic cytokine levels in mice. (**A**) Healthy C57BL/6J males received hCSF1-mFc (0–1.0 mg/kg, s.c.) once-daily for three consecutive days. Organs and biofluids were harvested and analysed 24 h after the final dose. Representative IHC stains versus F4/80 (murine macrophage marker) are shown in the micrographs for each indicated organ per treatment group. (**B**) Image analysis of F4/80 stains shows average percentage area (of F4/80-positive pixels) per treatment group for liver (left panel), spleen (middle panel), and kidney (right panel). (**C**) Scatter plots show individual plasma cytokine concentrations in treatment groups for IL-10 (left panel), IL-1β (center panel), and CXCL1 (right panel). (**D**) Scatter plots show individual liver function biomarker levels per treatment group quantified in plasma for albumin (left panel) and total bilirubin (right panel). (**E**) Scatter plots show cytokine levels quantified in individual liver homogenates for IL-12p70 (far left), IL-5 (center-left) (KW), IL-6 (centre-right) (KW), and TNFα (far right). Circles represent individual mice in charts. Clear circles – 0.0 mg/kg (vehicle alone); pale grey circles, 0.1 mg/kg; dark-grey circles, 0.5 mg/kg; black circles, 1.0 mg/kg. Shapiro-Walk normality test and ANOVA performed with Dunnetts post-hoc test unless otherwise stated. n.s., not significant; * *P* < 0.05; ** *P* < 0.01; *** *P* < 0.001. Abbreviations: IHC, immunohistochemistry; IL, interleukin; CXCL1, C-X-C Motif Chemokine Ligand 1; KW, Kruskal-Wallis test with Dunn’s multiple comparisons test TNFα, tumour necrosis factor alpha

Furthermore, short-term hCSF1-mFc treatment in human monocytes induced ERK phosphorylation at similar concentrations, indicating CSF1 receptor (CSF1R) stimulation (Fig. 1B). Next, a range of single hCSF1-mFc doses were administered to healthy male mice (0–1.0 mg/kg, s.c.; study design: Supporting Fig. S1A). A rapid body weight increase was observed in mice after hCSF1-mFc treatment up to 72 h, compared to vehicle controls (Supporting Fig. S1B). Blood sampling confirmed that circulating hCSF1 levels were detected in mice at ng/mL concentrations 24 h after injection demonstrating that hCSF1-mFc was not rapidly eliminated in vivo (Supporting Fig. S1C). However, no reduction in blood platelet levels (an anticipated response attributed to CSF1-induced splenomegaly) was observed in mice after any hCSF-mFC dose. Therefore, a repeat-dose study was performed in mice receiving three consecutive daily hCSF1-mFC administrations over a dose range (study design: Fig. 1C). Similar to the single-dose response, all hCSF1-mFC treated mice rapidly gained weight on average compared to vehicle controls animals, which experienced an overall loss due to daily tail vein venepuncture [32]. (Fig. 1D). Haematology showed a dose- dependent drop in blood platelet levels at 72 h indicating bioactivity in vivo after repeat hCSF1-mFc treatment (Fig. 1E). Organ weights revealed a dose-dependent increase in the liver- and spleen-to-body weight ratios 72 h after hCSF1-mFc treatment, whilst kidney and brain weight ratios remained unchanged (Fig. 1F). Furthermore, liver and spleen weight ratios increased with hCSF1-mFc dose if kidneys or brain weights were used as normalizers instead (Supporting Fig. S2). To investigate hepatic macrophage dynamics following repeat hCSF1-mFc treatment, immunohistochemical (IHC) techniques were performed in harvested organs. A striking dose-dependent increase in F4/80 positive cells (i.e. a murine macrophage marker) was observed after hCSF1-mFc treatment in liver tissue, resulting in a 3.7-fold increase after three 1.0 mg/kg administrations (Fig. 2A-B; antibody details provided in Supporting Table S1). Similar F4/80 increases were observed in spleen, but only a modest increase was observed in kidney, which reflected the respective changes in organ weight (Fig. 2B).

A dual IHC approach demonstrated that a significant proportion of liver macrophages were BrdU-positive (i.e. in S-phase) after pulse-labelling, indicating macrophage proliferation in situ (Supporting Fig. S3A, B). Since hCSF1-mFC treatment induced a striking increase in liver macrophages, a panel of cytokines and biomarkers were characterised in plasma and liver tissue. A prominent dose-dependent increase in concentrations of plasma IL-10, an anti-inflammatory/T-cell regulatory cytokine, was observed following hCSF1-mFc treatment, alongside concomitant decreases in proinflammatory IL-1β and CXCL1 plasma concentrations (Fig. 2C). Plasma albumin and bilirubin levels, two clinically-used circulating liver function biomarkers, showed a dose-dependent reduction in healthy mice after hCSF1-mFc treatment (Fig. 2D), although the plasma transaminase activity was not different (Supporting Fig. S3C). Further cytokine changes were recorded in whole liver homogenates with dose-dependent reductions in IL-12p70, IL-5, and IL-6, and a dose-dependent increase in TNFα (proinflammatory cytokine), which likely reflect the compositional changes in the liver by macrophages (Fig. 2E). Finally, endogenous murine CSF1 was quantified in the sera of healthy mouse treated with and without hCSF1-mFc to check if ectopic CSF1 delivery influenced endogenous levels, but no differences were observed between treatment groups (Supporting Fig. S4). Mice tolerated hCSF1-mFC administration well and no adverse events were observed.

### hCSF1-mFc treatment reduces serum proinflammatory cytokine levels following APAP-ALI

**Fig. 3:**
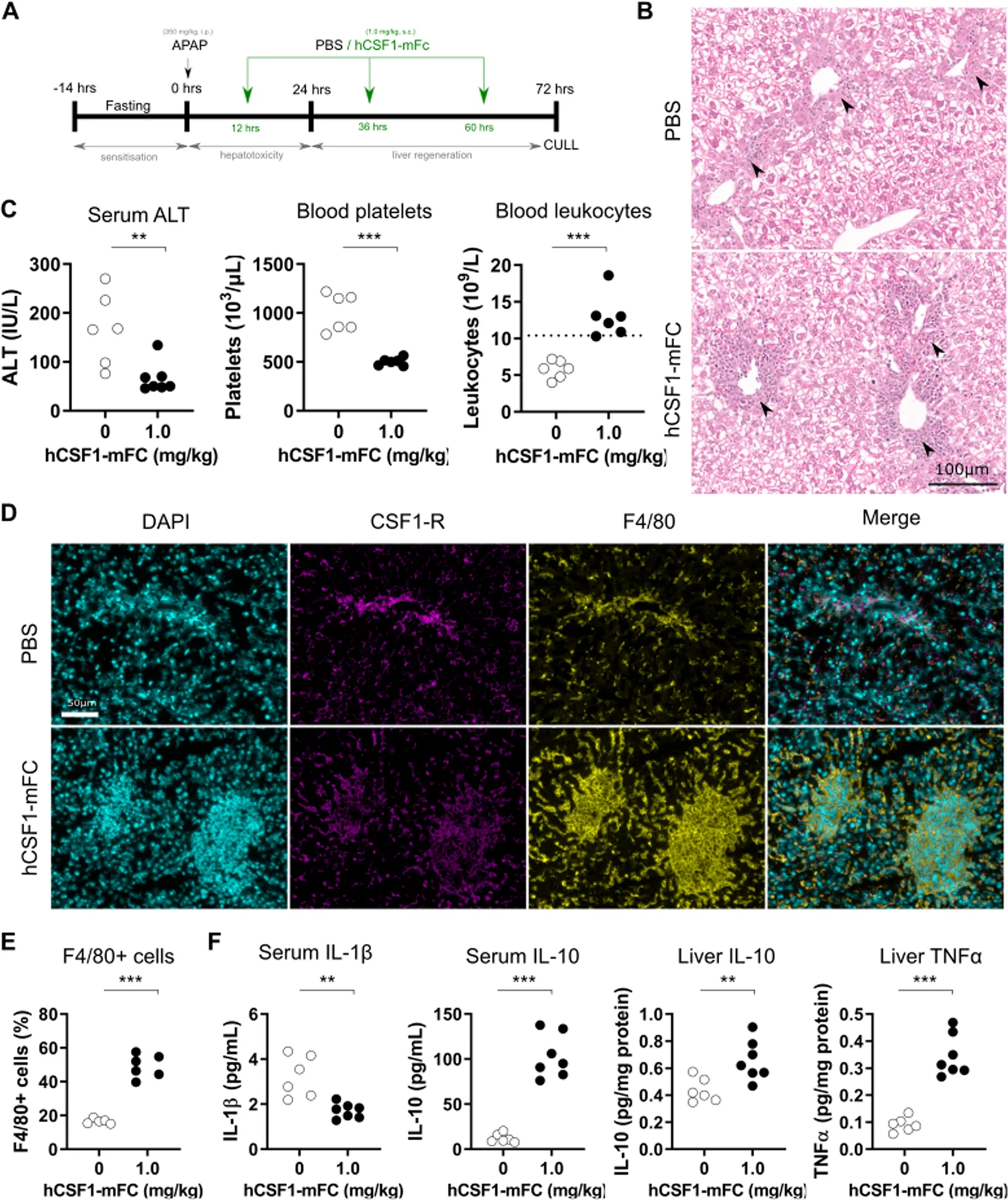
Iterative hCSF-mFc treatment reduces liver injury biomarkers and serum proinflammatory cytokines in APAP-ALI mice. (**A**) Study design: fasted C57BL6/J mice received APAP (350 mg/kg, i.p.) to induce ALI. APAP-ALI mice were treated with hCSF1-mFc or vehicle alone at 12, 36, and 60 h post-APAP, before tissue and biofluids were analysed at cull − 72 h. (**B**) Micrographs show representative H + E stains of liver tissue of APAP-ALI mice treated with vehicle (top) or hCSF1-mFc (bottom). Pronounced mononuclear cell infiltrate (black arrowheads) is observed at necrotic centrilobular regions in the liver of hCSF1-mFc treated mice. (**C**) Scatter plots show individual Serum ALT (MW), Blood platelets and leukocytes. (**D**). IF stains show higher numbers of cells dual positive for F4/80 and CSF1R at necrotic regions in hCSF1-mFc treated mice, indicating macrophage accumulation. (**E**) Image analysis shows mean percentage of F4/80-positive cells quantified in IF-stained liver sections for individual mice displayed in treatment groups. (**F**) Cytokines were quantified in serum and liver homogenates of APAP-ALI treated with hCSF1-mFc or vehicle alone. Serum IL-1β (far left panel) and serum IL-10 (centre-left panel) concentrations are shown in treatment groups. Hepatic IL-10 (centre-right), and hepatic TNFα levels (MW) (far right panel) are also shown. Circles represent individual mice in charts. Clear circles – 0.0 mg/kg (vehicle alone); black circles, 1.0 mg/kg. Shapiro-Walk normality test performed for all, Unpaired t-test performed unless otherwise stated. MW, Mann-Whitney test. n.s., not significant; ** *P* < 0.01; *** *P* < 0.001. Abbreviations: ALT, alanine aminotransferase; APAP, acetaminophen; IL, interleukin; PBS, phosphate-buffered saline; TNFα, tumour necrosis factor alpha

Since hCSF1-mFC treatment appeared safe and induced a systemic anti-inflammatory response in healthy mice, we hypothesised that hCSF1-mFc may have therapeutic value in an acute inflammatory model. A dose of 1 mg/kg was selected as this was well tolerated in healthy mice and showed biological activity with platelet reduction, indicating engagement of Fc‑dependent immune functions, alongside confirming increased macrophage numbers. The acetaminophen (APAP)-induced acute liver injury (APAP-ALI) model was selected due to its translational value. Therefore, hCSF1-mFc (or vehicle alone) was administered repeatedly to APAP-ALI mice (study design: Fig. 3A). Histological examination of liver tissue at 72 h showed evidence of centrilobular necrosis in APAP-ALI mice with a pronounced cellular infiltrate within necrotic areas in hCSF1-mFc treated mice (Fig. 3B). Importantly, hCSF1-mFC treated mice had lower serum ALT activity versus PBS-treated controls (Fig. 3C). Similar to effects in healthy animals, blood platelets were lower in hCSF1-mFc treated APAP-ALI mice indicating a conserved bioactivity in this model (Fig. 3C). In addition, APAP-ALI mice treated with vehicle alone displayed moderate leukopenia, however hCSF1-mFc treatment restored blood leukocyte counts to normal healthy levels (Fig. 3C). Dual immunofluorescent (IF) staining in liver tissue showed increased levels of cells positive for CSF1 receptor and F4/80 (i.e. macrophages), particularly at necrotic sites, which was approximately three-fold higher in hCSF1-mFC mice (Fig. 3D). Serum IL-10 was 8.5-fold higher in CSF1-mFC treated APAP-ALI mice compared to mice treated with vehicle alone. Conversely, serum IL-1β levels were 47% lower in hCSF1-mFc treated mice compared to vehicle controls. Finally, cytokine analysis in whole liver homogenate also showed a 49% increase in IL-10 levels following hCSF1-mFc treatment, whereas TNFα levels were 3.8-fold higher, similar to effects in healthy animals. Terminal deoxynucleotidyl transferase dUTP nick end labelling (TUNEL) was performed in liver sections to identify necrotic/apoptotic cells, revealing a trend towards a reduced frequency in hCSF1-mFc treated mice (Supporting Fig. S5). Together, these results suggest hCSF1-mFc treatment induces a systemic anti-inflammatory response in experimental APAP-ALI.

### hCSF1-mFc treatment improves hepatic phagocytic capacity during liver fibrosis

**Fig. 4:**
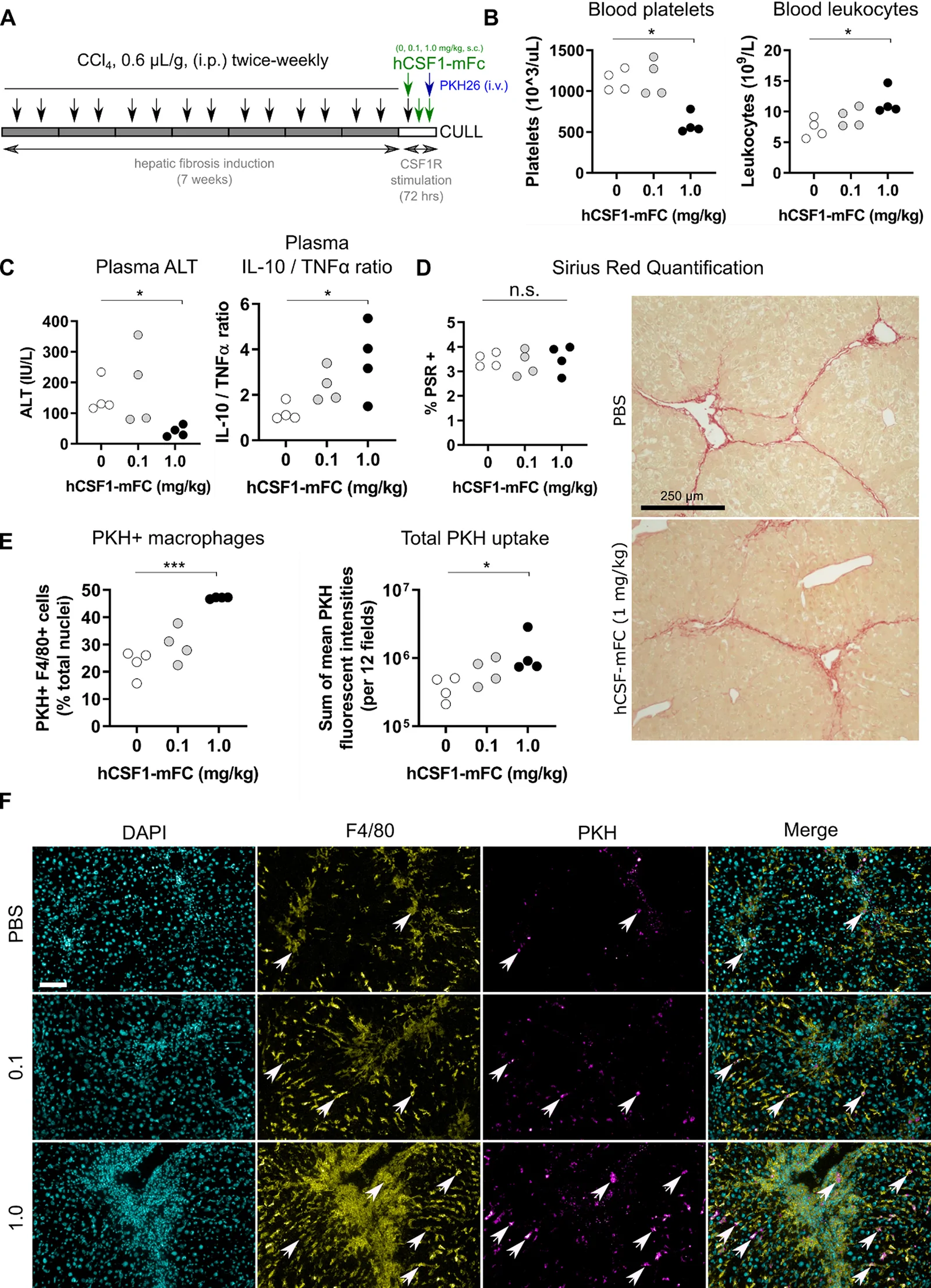
Iterative hCSF-mFc treatment increases phagocytic capacity during experimental liver fibrosis in mice. (**A**) Study design: liver fibrosis was established in C57BL/6J mice by twice-weekly CCl_4_ injection for 7 weeks. Fibrotic mice then received daily administrations of hCSF1-mFC (0.1–1.0 mg/kg, s.c.), or vehicle alone, for three consecutive days. All mice received PKH26, a soluble phagocytic label, 24 h before cull. (**B**) Hematology data shows whole blood counts for platelets (left panel) and leukocytes (right panel) in fibrotic mice treated with or without hCSF1-mFc. (**C**) Plasma biomarker data shows plasma ALT activity (KW) (left) and plasma IL-10/TNFα ratio (right) in fibrotic mice in treatment groups. (**D**) Picrosirius red stains were performed in fixed liver sections. Representative micrographs are provided for corresponding treatment groups (right). Quantification from image analysis is shown (left). (**E**) PKH uptake is quantified in the percentage of PKH-positive F4/80-positive cells (left), and total PKH fluorescent intensity in F4/80-positive cells, averaged per field (right). (**F**) IF images show representative staining for DAPI (cyan, far-left), F4/80 (yellow, center-left), and PKH (magenta, centre-right), with merged images (far-right) taken from fixed liver sections in indicated treatment groups. White arrowheads indicate cells dual positive for F4/80 and PKH. Circles represent individual mice in charts. Clear circles – 0.0 mg/kg (vehicle alone); pale-grey circles, 0.1 mg/kg; black circles, 1.0 mg/kg. Shapiro-Walk normality test and ANOVA performed with Dunnetts post-hoc test unless otherwise stated. n.s., not significant; * *P* < 0.05; *** *P* < 0.001. Abbreviations: ALT, alanine aminotransferase; CCl_4_, carbon tetrachloride; DAPI, 4′,6-diamidino-2-phenylindole; IL, interleukin; PBS, phosphate-buffered saline; PKH (26-PCL), Paul Karl Horen (26-phagocytic cell labelling); TNF, tumour necrosis factor alpha

**Fig. 5:**
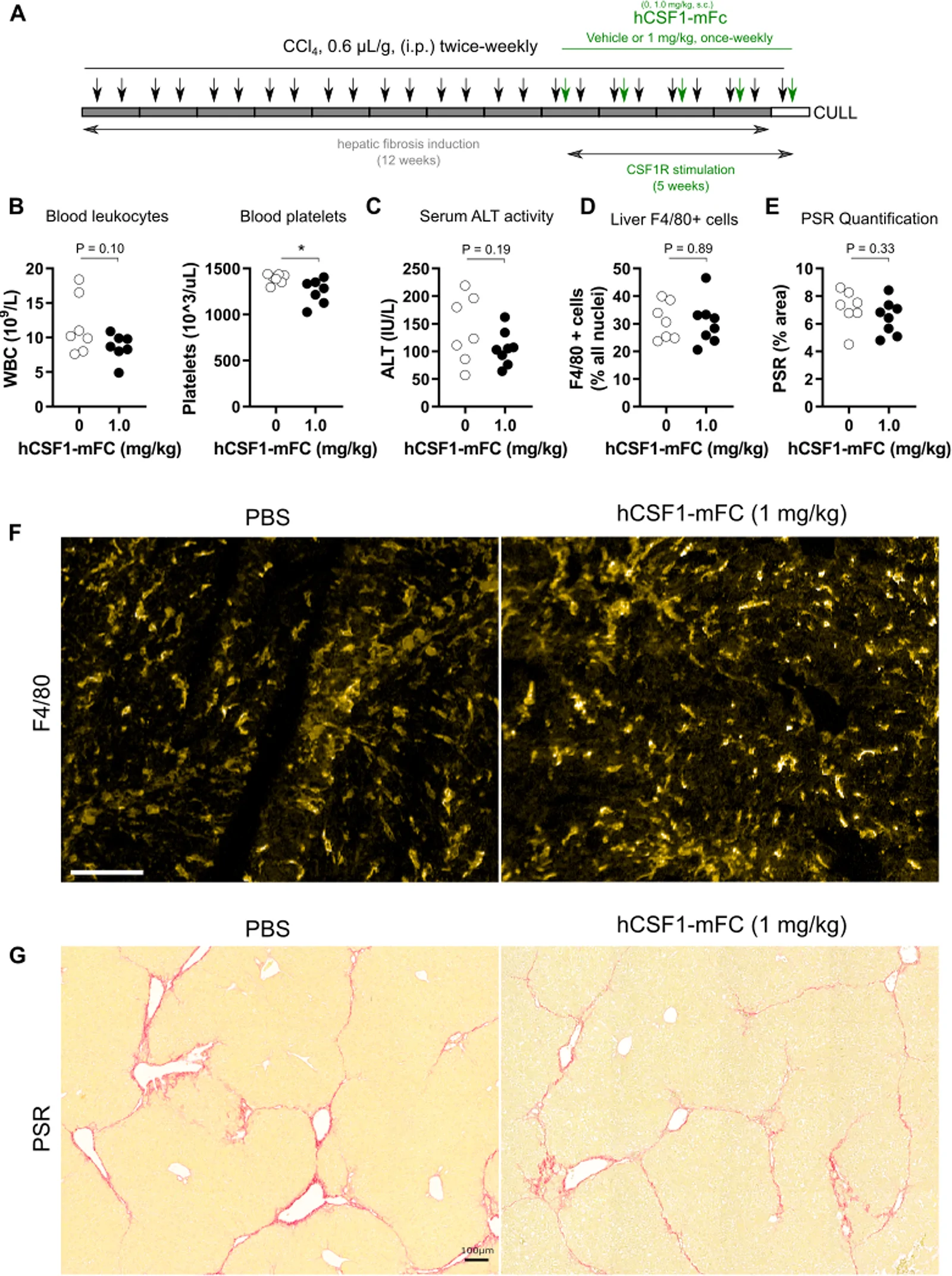
Once-weekly hCSF1-mFc administration during a 12-week iterative CCl_4_-induced fibrosis model does not change hepatic macrophage levels or liver fibrosis. (**A**) Study design: Liver fibrosis was established using an iterative 12-week CCl4 model (0.6 L/g; 1:4 olive oil, i.p.). Fibrotic mice were randomized to receive hCSF1-mFc (1 mg/kg, s.c.) or vehicle alone as treatments, once-weekly, from weeks 9 through 12, inclusive, with a final treatment at the start of the 13th week. (**B**) Hematology data shows final blood leukocytes (left) and blood platelet counts (right) in treatment groups. (**C**) Final Serum ALT activity in treatment groups. (**D**) Hepatic F4/80 cells are quantified as percentage of total liver cells, from IF-based imaging and shown in treatment groups. (**E**) Quantification of PSR staining from image analysis is shown per treatment group. (**F**) Representative IF photomicrographs of F4/80 staining are shown for vehicle-treated (left) hCSF1-mFc-treated (right) fibrotic mice. (**G**) Representative photomicrographs of PSR staining are shown for vehicle-treated (left) hCSF1-mFc-treated (right) fibrotic mice. Shapiro-Walk normality test and Unpaired t-test performed. * *P* < 0.05, other *P* values provided. Each circle represents an individual mouse. Abbreviations: ALT, alanine aminotransferase; CCl4, carbon tetrachloride; PBS, PSR, picrosirius red; phosphate-buffered saline; WBC, white blood cells

Since hCSF1-mFc treatment invoked a predominantly anti-inflammatory response in an acute injury model, and as porcine CSF-1-mFc improved thiocetamide (TAA) hepatic fibrosis [27] we tested whether hCSF1-mFc treatment could improve hepatic innate immune performance in fibrosis, where serious complications arise from hepatic immune deficiency [9, 11]. After seven weeks of CCl_4_ treatment (0.6 uL, twice weekly, i.p.), mice received iterative treatment with hCSF1-mFc (0.1–1.0 mg/kg, s.c.) or vehicle alone and were culled 24 h later. Alongside the final hCSF1-mFc (or vehicle) administration, all mice received a fluorescent soluble dye that is phagocytosed and thereby specifically labels phagocytic cells (PKH26PCL, i.v; study design: Fig. 4A). At cull, hCSF1-mFc bioactivity was evidenced in this model by reduced blood platelets and increased blood leukocytes in fibrotic mice receiving hCSF1-mFc (1.0 mg/kg), as previous (Fig. 4B). Similar to the acute model, plasma ALT was lower in fibrotic mice receiving hCSF1-mFC (1.0 mg/kg) compared to vehicle alone (Fig. 4C). In addition, we calculated the ratio between IL-10 (anti-inflammatory cytokine) and TNFα (proinflammatory cytokine) in plasma. hCSF1-mFc treated mice displayed a higher IL-10/TNFα ratio at 1.0 mg/kg indicating an anti-inflammatory response (Fig. 4C). Hepatic collagen content was equivalent between treatment groups as quantified by picrosirius red staining (Fig. 4D). Since extracellular matrix remodelling is not a rapid process, we designed a separate 12-week CCl_4_ fibrosis experiment to test if hCSF1-mFc dosed once-weekly over the final five weeks could elicit collagen regression (Fig. 5A). However, once-weekly hCSF1-mFc dosing was insufficient to alter hepatic macrophage levels or collagen content (Fig. 5D-G) demonstrating a requirement for at least twice weekly dosing to impact fibrosis [27]. In the seven-week fibrosis study, hepatic phagocytic capacity was quantified by PKH26PCL (herein referred to as PKH) epifluorescence in F4/80-stained IF sections. PKH26PCL was injected i.v. 24 h prior to cull, and particulates are only up taken by phagocytic cells. A 2.1-fold increase in the number of PKH-positive F4/80-positive cells, plus a 3.5-fold increase in total PKH signal intensity was observed after hCSF1-mFc treatment (1.0 mg/kg), compared to fibrotic mice receiving vehicle alone (Fig. 4E, F), showing increased phagocytosis in hepatic macrophages. In the same study, enzymatic digestion was performed on fibrotic liver tissue to retrieve the hepatic leukocyte population with and without hCSF1-mFC treatment. A flow cytometry gating strategy was used to identify resident (F4/80^hi^ CD11b^int^) and infiltrating macrophage (F4/80^int^ CD11b^hi^) populations (Supporting Fig. S6). hCSF1-mFC (1.0 mg/kg) treatment had the most striking effect on the infiltrating macrophage population showing an increased frequency of PKH-positive cells and mean fluorescent intensity (MFI), although the resident population also showed a higher MFI trend, but did not reach statistical significance (Supporting Fig. S7A), consistent with the findings of Keshvari et al., that both infiltrating and resident macrophages increase in number following CSF1-Fc. Finally, Ly6C, a macrophage phenotypic surface marker, was measured in hepatic macrophage populations. Ly6C is predominantly expressed on infiltrating macrophages (particularly proinflammatory populations), which was reflected by the MFIs in hepatic macrophage subsets (Supporting Fig. S7B). Importantly, hCSF1-mFc treatment did not change Ly6C status in infiltrating macrophages providing further evidence hepatic macrophages do not adopt a deleterious inflammatory phenotype post-treatment.

### hCSF1-mFc treatment induces hepatocyte proliferation in healthy and liver disease models

**Fig. 6:**
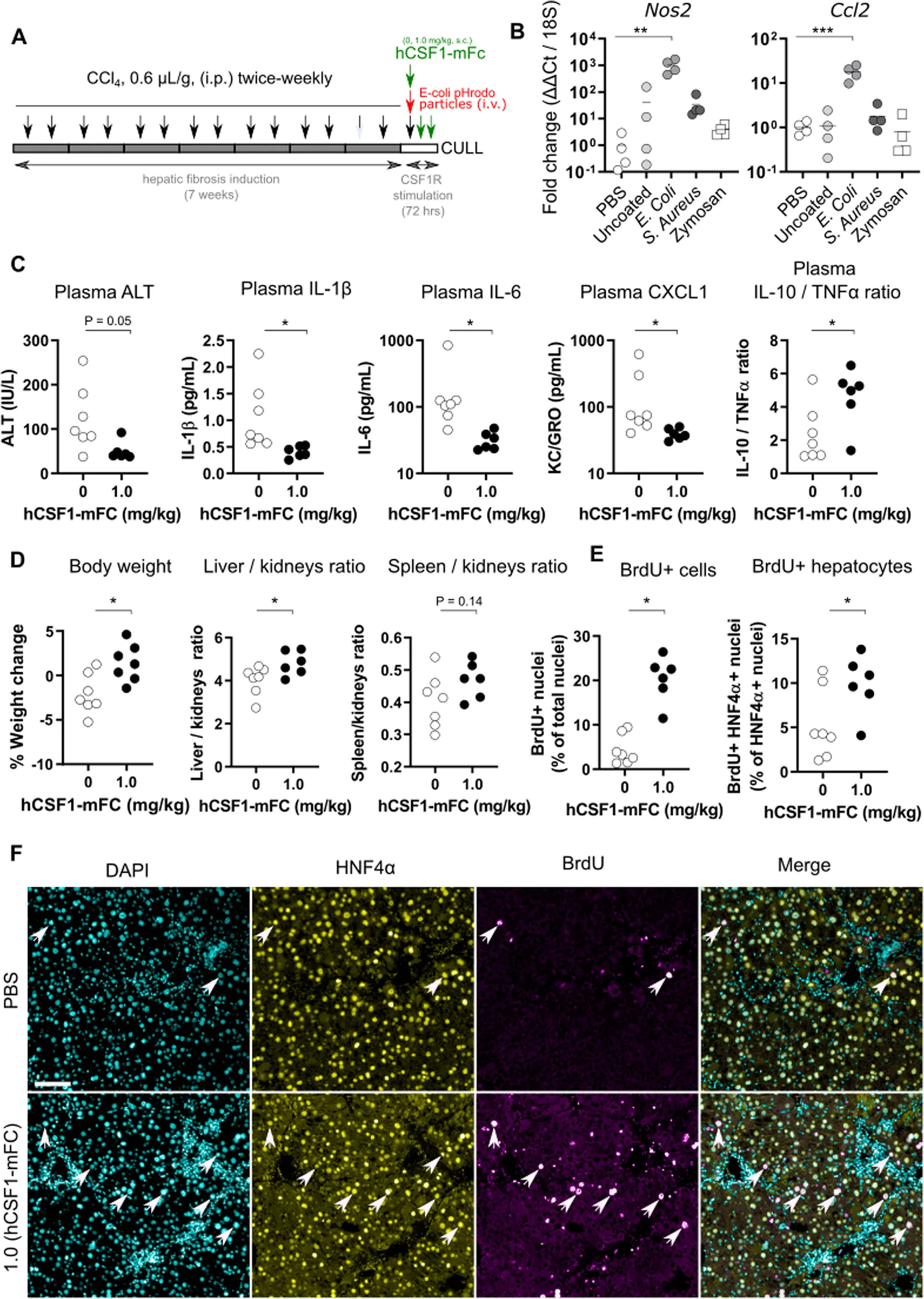
Iterative hCSF-mFc treatment reduces circulating proinflammatory cytokines and promotes hepatocyte proliferation in co-stimulated fibrotic mice. (**A**) Study design: the seven-week CCl_4_ fibrosis protocol was modified to include a single administration of *E. coli*-coated bioparticles (i.v.) alongside the final CCl_4_ injection. Mice then received daily treatments with hCSF1-mFc or vehicle alone for three consecutive days. All mice received BrdU (1 mg, i.p.) one hour before cull. (**B**) RT-PCR data shows gene expression for pro-inflammatory genes Nos2 and Ccl2 in BMDMs indicating a specific pro-inflammatory response in *E. coli*-coated bioparticles. Gene expression data is calculated using the 2^^−ΔΔCt^ method and *18 S rRNA* served as the housekeeping gene. (**C**) Scatter plots show plasma chemistry data for ALT (MW), IL-1β, IL-6 (MW), CXCL1 (MW), and the plasma IL-10/TNF ratio (left to right) in indicated treated groups. (**D**) Scatter plots show % body weight change (left panel), liver/kidneys weight ratio (center), and spleen/kidneys weight ratio (right) in indicated treatment groups. (**E**) BrdU-positive nuclei were calculated from IF images and expressed as a percentage of total nuclei (left panel) or dual BrdU- HNF4α-positive nuclei as a percentage of total HNF4α-positive nuclei (MW) (right panel). (**F**) IF images show representative staining for DAPI (cyan, left), HNF4α (yellow, centre-left), and BrdU (magenta, centre-right) with merged image shown (far-right). White arrows indicate nuclei positive for HNF4α and BrdU. Circles represents individual mice in charts. Clear circles – 0.0 mg/kg (vehicle alone); pale-grey circles, 0.1 mg/kg; black circles, 1.0 mg/kg. Shapiro-Walk normality test and Unpaired t-test performed unless otherwise stated. MW, Mann-Whitney test * *P* < 0.05; ** *P* < 0.01; *** *P* < 0.001. Abbreviations: ALT, alanine aminotransferase; BrdU, 5-bromo-2’-deoxyuridine; CCl_4_, carbon tetrachloride; CXCL1, C-X-C Motif Chemokine Ligand 1; DAPI, 4′,6-diamidino-2-phenylindole; HNF4α, Hepatocyte Nuclear Factor 4 alpha; IL, interleukin; PBS, phosphate-buffered saline; PKH(26-PCL), Paul Karl Horen (26-phagocytic cell labelling dye); TNFα, tumour necrosis factor alpha

To further profile the anti-inflammatory effects of hCSF1-mFc treatment, we modified the chronic CCl_4_ model by co-administering a bolus dose of a pro-inflammatory agent after fibrosis was established, to better model an acute-on-chronic injurious event (study design: Fig. 6A). Several commercially-available reagents are available, which mimic noxious infectious agents, including an array of pH-dependent fluorescently-labelled bioparticles (pHrodo^®^) coated with a selection of bacterial/fungal-derived substances. First, we tested if these coated bioparticles could provoke a proinflammatory response in murine bone-marrow derived macrophages (BMDMs) in vitro. Gene expression profiling showed that only *E. coli*-coated bioparticles substantially increased *Nos2* and *Ccl2* expression in BMDMs, i.e. two genes associated with classical-activation and a proinflammatory response (Fig. 6B, primer details provided in Supporting Table S2). Next, *E. coli*-coated bioparticles were administered in vivo to test if they could elicit an inflammatory response in liver tissue after intravenous delivery (400 µg, i.v.) to healthy mice. Eight hours post-treatment, *E. coli* coated bioparticle-treated mice displayed weight loss, had reduced serum albumin, and showed a striking hepatic neutrophil infiltration compared to vehicle controls, consistent with a proinflammatory response (Supporting Fig. S8A-D). Importantly, *E. coli* bioparticles were colocalised with F4/80 cells in liver sections demonstrating that these bioparticles are engulfed by hepatic macrophages after i.v. administration (Supporting Fig. S8E). Next, hCSF1-mFc was tested using the repeat administration regimen as a treatment in the modified *E. coli-*pHrodo/CCl_4_ model. A lower trend for plasma ALT activity (*p* = 0.05) was observed after hCSF1-mFc treatment (Fig. 6C). More strikingly, hCSF1-mFc treatment led to reductions in a panel of plasma proinflammatory markers including IL-1β, IL-6, and CXCL1, and led to an increase in the IL-10/TNFα ratio indicating an anti-inflammatory response (Fig. 6C and Supporting Fig. S9). Gross observations showed that hCSF1-mFc treatment attenuated weight loss and led to an increase in the liver/kidneys weight ratio (Fig. 6D). Gene expression was higher for *Mmp8* and *Mmp9*, which encode two collagenolytic proteases, was observed in whole liver tissue preparations of hCSF1-mFc treated mice, although *Mmp13* expression was not different (Supporting Fig. S9). Acta2 and Mrc1 expression were higher in injured livers following hCSF1-mFc, indicating increased abundance of CD206 + macrophages linked to matrix remodelling [33]. Arg1 conversely was reduced, which may reflect changes in macrophage populations [34].

**Fig. 7:**
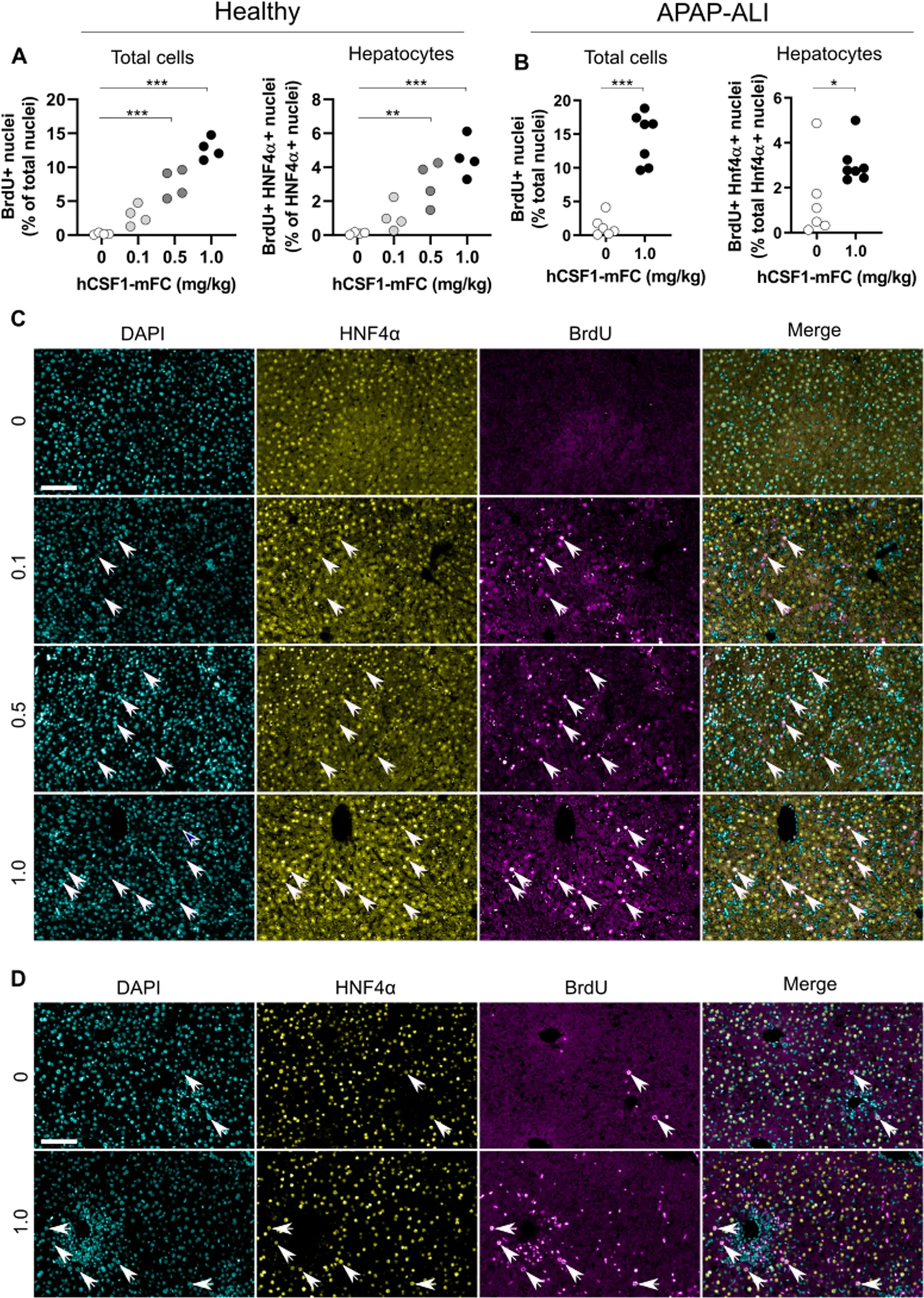
Iterative hCSF1-mFc treatment induces BrdU incorporation in hepatocytes in healthy and APAP-ALI mice. (**A**) Scatter plots shows quantification of BrdU incorporation (percentage BrdU-positive nuclei) in treatment groups from healthy mice treated with hCSF1-mFC (0.1–1.0 mg/kg, s.c.) or vehicle alone. Left panel shows total BrdU incorporation in all nuclei. Right panel shows BrdU incorporation in HNF4α-positive cells only (ANOVA). (**B**) Scatter plots shows quantification of BrdU incorporation (percentage BrdU-positive nuclei) in treatment groups from APAP-ALI mice treated with hCSF1-mFC (1.0 mg/kg, s.c.) or vehicle alone. Left panel shows total BrdU incorporation in all nuclei (t-test). Right panel shows BrdU incorporation in HNF4α-positive cells only (MW). (**C**). Representative IF photomicrographs of liver tissue harvested from healthy mice treated with hCSF1-mFc or vehicle alone. Panels show fluorescent channels for DAPI (cyan, nuclear marker, left) HNF4α (yellow, hepatocyte-marker, centre-left), BrdU (magenta, proliferation marker, centre-right), and overlay merged channel (right). Panels shown in treatment groups (0–1.0 mg/kg, in rows). Arrowheads indicate nuclei dual positive for HNF4α and BrdU. (**D**) Representative IF stains from APAP-ALI mice in fluorescent channels, as above. Panels presented in treatment groups for APAP-ALI mice receiving vehicle alone (top row), or hCSF1-mFc (1.0 mg/kg, s.c.; bottom row). * < 0.05, ** *P* < 0.01, *** *P* < 0.001. Abbreviations: APAP-ALI, acetaminophen-induced acute liver injury; BrdU, 5-bromo-2’-deoxyuridine; DAPI, 4′,6-diamidino-2-phenylindole. HNF4α, hepatocyte nuclear factor 4 alpha. MW, Mann Whitney test

To better understand the regenerative response in the liver following hCSF1-mFc treatment, we analysed HNF4α/BrdU dual IF labelling to quantify cell proliferation post-treatment. We observed a striking increase in the frequency of BrdU-positive cells in the *E. coli* stimulated fibrotic liver following hCSF1-mFc treatment (Fig. 6E). Furthermore, there was a significant increase in the frequency of BrdU-positive nuclei in HNF4α-positive cells suggesting hCSF1-mFc treatment specifically promotes hepatocyte regeneration, in addition to alleviating inflammation (Fig. 6E). Using the same technique, increased BrdU incorporation in HNF4α-positive cells was also confirmed after hCSF1-mFC treatment in both healthy and APAP-ALI mice demonstrating a conserved response across several models (Fig. 7).

Since hCSF1-mFC treatment induced a robust proliferative response in hepatocytes, we postulated whether there was a reduction in cellular senescence occurred in these disease models. p21 is a p53‑induced CDK inhibitor upregulated in senescent cells, which is widely used as a senescence marker. Therefore, we measured nuclear p21 intensity in fibrotic mice with and without hCSF1-mFc treatment, but observed no difference between treatment groups (Supporting Fig. S10). Interestingly, a dose-dependent increase in nuclear p21 staining was observed following hCSF1-mFc treatment in both healthy and APAP-ALI mice (Supporting Fig. S11) – i.e. the same groups where a striking proliferative response was observed. It is possible that after hCSF1-mFc treatment, senescence and proliferation are occurring in tandem or that p21 may be acting as a assembly factor [35].

## Discussion

Hepatic macrophage depletion occurs during acute and chronic liver disease leading to perturbations in barrier function and compromised clearance capacity [36]. Consequently, patients with acute and chronic liver disease are at risk of developing infections, typically *E. coli* or candidiasis, which are major determinants of death or delisting for transplant [37, 38]. Novel therapeutic strategies are required to potentially restore barrier function, improve hepatic innate immunity, and stimulate healthy parenchymal regeneration to improve clinical outcomes. Previous studies using animal-derived CSF1-Fc fusion proteins have increased our understanding of macrophage dynamics in experimental models of health and liver disease [24–27, 39]. Herein, we conjugated the bioactive amino acid sequence of hCSF1 to the Fc region of mouse or human immunoglobulin to yield fusion proteins suitable for in vivo testing in experimental models of liver disease.

hCSF1-mFc treatment induced a robust and reproducible increase in tissue macrophages, especially in mouse liver, which was conserved in all murine disease models. Human and mouse CSF1 proteins share high sequence homology, especially in the N-terminal domain, which confers CSF1R binding activity [40, 41]. As such, hCSF1 is active in the mouse [42]. There are several potential advantages of hCSF1-Fc protein therapy versus macrophage cell therapy for liver diseases and these include but are not limited to; the improved logistics of provision and lower cost, broad tuneable stimulation of endogenous macrophages and increased safety considerations, where adverse effects can be managed by dose interruption. Our fusion proteins contained the bioactive sequence only (amino acids 36–190) of hCSF1, which is 82% homologos to the corresponding mouse sequence. Native CSF1 is rapidly cleared from the circulation predominantly via receptor-mediated endocytosis by KCs leading to a half-life of only 1.6 h in mice [23]. Previously, Fc conjugation of animal CSF1 improved circulating half-life by increasing the molecular weight to prevent renal loss of residual circulating CSF1 [24]. Here, the same strategy was employed using hCSF1. We detected hCSF1 levels in mice for at least seven days after a single administration suggesting the fusion protein is not rapidly cleared in vivo. hCSF1-mFc administration was well-tolerated, reduced plasma CXCL1 and IL-1β levels, and led to a striking increase in plasma IL-10 concentrations. IL-10 is a regulatory T cell (Treg) cytokine and has intrinsic immunosuppressive activity *via* the inhibition of proinflammatory cytokine responses. Moreover, IL-10-producing macrophages have been found to be efficient at clearing apoptotic material [43]. Increasing systemic IL-10 levels after hCSF1-mFc indicates strongly activated regulatory/anti‑inflammatory pathways and may be secondary to macrophage production. This could represent a major therapeutic mechanism of hCSF-mediated therapy, especially in liver diseases where uncontrolled inflammation (e.g. Systemic inflammatory response syndrome [SIRS]) can be lethal. In the APAP-ALI model, hCSF1-mFc treatment attenuated systemic IL-1β concentrations, a key proinflammatory cytokine that potentiates the inflammatory response and injury during APAP-ALI [44]. In fact, systemic IL‑1β also decreased following hCSF1-mFc in co-stimulated fibrotic mice, which is likely secondary to expansion and reprogramming of hepatic macrophages to reparative phenotypes. This coincided with reduction of tissue injury, measured by serum ALT activity, a clinically-used ALI biomarker. Earlier assessment is likely to have identified reduction in necrosis with hCSF1-mFc, as this proceeds changes in ALT. hCSF1-mFc treatment also caused a dose-dependent reduction in plasma albumin in healthy mice. Macrophages scavenge a range of serum proteins [45], including albumin [46], which is used as a biomarker of hepatocyte function. Vigilance should be exercised when interpreting protein-based biomarkers in liver disease models when clearance mechanisms are targeted.

Importantly, hCSF1-mFc treatment increased PKH26PCL uptake (a soluble dye that labels cells performing phagocytosis) in the CCl_4_ fibrosis model. This finding demonstrates that hCSF1-mediated macrophage accumulation increases hepatic clearance capacity during liver fibrosis. Chronic liver disease patients are at risk of developing enteric infections associated with pathological bacterial translocation resulting from increased intestinal permeability and hepatic barrier failure. Consequently, infections are common in cirrhotic patients and confer a 3.75-fold increased risk of death [9, 36, 38]. Increasing numbers of resident and infiltrating macrophages to restore barrier function, *via* CSF1-Fc treatment, might be an attractive therapeutic strategy in chronic liver disease patients. However, further work is required in infection models to confirm if CSF-Fc therapy is safe and effective to prevent or control sepsis.

Fibrosis was not reduced by once weekly administration in this CCl_4_ fibrosis model indicating more frequent administration is required. Previously, increased hepatocyte proliferation was observed after porcine CSF1-Fc treatment in healthy mice [24] and in TAA fibrosis [27]. Here, we report CSF1-Fc treatment induced hepatocyte proliferation in fibrosis and in ALI models. The mechanism that underpins hCSF1-induced hepatocyte proliferation is not known, but trophic action by hepatic macrophages has been speculated, such as secreting growth factors and cytokines (e.g. HGF, IL‑6, TNF‑α, VEGF) and remodelling the microenvironment. We observed a dose-dependent increase in hepatic TNFα levels in liver homogenates from hCSF-mFc-treated mice. TNFα drives hepatocyte proliferation following partial hepatectomy [47]. However, other macrophage-derived molecules such as hepatocyte growth factor have also been suggested [39, 48] and found increased with increased with CSF1 [27]. Hepatocyte proliferation was also observed after alternatively-activated macrophage cell therapy in ALI models [17], which suggests that proliferative signals are macrophage-derived rather than directly attributed to CSF1R stimulation. Hepatocyte proliferation is fundamentally required for the restoration of healthy liver parenchyma during regeneration, and represents a key mechanism in hCSF-Fc therapy. Future studies may be indicated in chronic fibrosis models to certify a lack of impact on carcinoma progression, as CSF1 inhibitors have anti-tumour activity [49]. Consideration could be given to the use of combination therapies, such as hCSF1‑Fc paired with NK‑cell infusions to provide direct cytotoxicity against tumour cells alongside regeneration, building on emerging NK‑cell–based strategies in HCC [50]. Alternatively, with anti‑fibrotic agents such as Oltipraz or other TGF‑β/stellate‑cell–targeting drugs [51] so that pharmacologic suppression of new matrix deposition is coupled with an expanded pool of pro‑resolving macrophages to accelerate extracellular‑matrix degradation and liver repair. One limitation with hCSF1-mFc treatment was the presence of dose-dependent thrombocytopenia likely associated with increased splenic sequestration of platelets. Although reduced blood platelets caused no adverse events in mice, it might represent a hurdle for clinical translation. This could be addressed by prophylactic platelet monitoring with set interventions, such as treatment breaks or dose fractioning, at clinically relevant platelet reductions.

## Conclusions

We report the production of a novel hCSF1 fusion protein, that robustly increases hepatic macrophage populations in models of acute and chronic liver disease. Consequently, hCSF1-mFc treatment improved hepatic clearance capacity during fibrosis, reduced circulating proinflammatory cytokines during fibrosis and ALI, and stimulated hepatocyte proliferation to promote liver regeneration. This report provides further evidence to facilitate the translation of CSF1-based fusion proteins towards clinical use.

## Supplementary Information

Below is the link to the electronic supplementary material.

Supplementary Material 1

## Data Availability

All data supporting the findings of this study are available within the paper and its Supplementary Information.
