# Supplementary material for: Administration of human macrophage colony-stimulating factor-mFc fusion protein increases macrophage phagocytic capacity and stimulates liver regeneration in mice
Source: J Inflamm (Lond). 2026 Jun 25;23:27. doi: 10.1186/s12950-026-00507-8 (PMC13673622; doi:10.1186/s12950-026-00507-8)
Supplement: Supplementary file 1 [file 12950_2026_507_MOESM1_ESM.docx]

**Administration of human macrophage colony-stimulating factor-mFc fusion protein increases hepatic phagocytic capacity and stimulates liver regeneration in mice**

Philip J. Starkey Lewis, Jennifer A. Cartwright, Lara Campana, Birgit U. Baumgarten, Hilmar Ebersbach, Marie-Gabrielle Ludwig, Gregory Marszalek, Benjamin Stutchfield, Rhona Aird, Tak-Yung Man, Eoghan O’Duibhir, Klaus Seuwen, Stuart J. Forbes

Table of contents

Supporting Fig. S1…………………………………………………………………………2

Supporting Fig. S2…………………………………………………………………………3

Supporting Fig. S3…………………………………………………………………………4

Supporting Fig. S4…………………………………………………………………………5

Supporting Fig. S5…………………………………………………………………………6

Supporting Fig. S6…………………………………………………………………………8

Supporting Fig. S7…………………………………………………………………………9

Supporting Fig. S8…………………………………………………………………………11

Supporting Fig. S9…………………………………………………………………………13

Supporting Fig. S10 ……………………………………………………………………….14

Supporting Fig. S11 ………………………………………………………………….……15

Supporting Table S1……………………………………………………………………….17

Supporting Table S2……………………………………………………………………….18

Supporting methods ……………………………………………………………………….19

Supporting references ……………………………………………………………………..25

**Supporting Figures**

Supporting Fig. S1. hCSF1-mFc is detectable in mouse plasma for 7 days post-administration. (A) Study design: Healthy 6 week old C57Bl/6J males received a single administration of hCSF1-mFc (0.0 – 1.0 mg/kg, s.c.). Plasma was extracted from whole blood collected *via* venesection at 1, 3, and 5 days after hCSF1-mFc administration, before cull at 7 days. (B) Daily average percentage body weight changes shown in treatment groups. (C) hCSF levels were quantified in plasma. Averages displayed per treatment group around the standard error of the mean. (D) Average blood platelet counts do not show hCSF1-induced thrombocytopenia after a single administration. Charts display mean ± standard deviation, unless stated otherwise.

Supporting Fig. S2. Iterative hCSF1-mFc treated shows a dose-dependent increase in liver and spleen weight ratios, regardless of normalization strategy. (A). Scatter plots show individual organ weight ratios in treatment groups standardized to individual brain weights. (B). Scatter plots show individual organ weight ratios in treatment groups standardised to individual kidney weights. * *P* < 0.05; ** < *P* < 0.01; *** *P* < 0.001.

Supporting Fig. S3. Iterative hCSF1-mFc treatment promotes BrdU incorporation in hepatic F4/80-positive cells in healthy mouse liver. (A) Dual-IHC stains show membranous F4/80 staining (Vector Red, magenta stain) and nuclear BrdU incorporation (DAB, brown stain) in vehicle-treated (left panel) and hCSF1-mFc-treated (right panel) mice. Dual isotype control stain provided (bottom right). Haematoxylin served as the counterstain. (B) Percentage breakdown of total hepatic cells based on F4/80 and BrdU staining for mice treated with hCSF1-mFC or vehicle alone. (C). Scatter plots show plasma ALT (left panel) and AST (right panel) activity mice in treatment groups after hCSF1-mFc administration in healthy mice. n.s., not statistically significant.

Supporting Fig. S4. Iterative hCSF1-mFc treatment does not influence systemic murine CSF1 levels in healthy or liver disease mouse models. (A). Immunosorbant assay specifically detects murine CSF1. Standard curves of mouse recombinant CSF1 (mrCSF1, dark grey), recombinant human CSF1 (hrCSF1, pale grey), or hCSF1-mFc (black) were assayed on an immunosorbant mouse CSF1 test kit. (B) Murine CSF1 levels were detected in plasma of healthy mice treated with hCSF1-mFc (0.1 - 1.0 mg/kg, s.c.) or vehicle alone. (C) Murine CSF1 levels were detected in plasma of CCl_4_-induced fibrotic mice treated with hCSF1-mFc (0.1 - 1.0 mg/kg, s.c.) or vehicle alone. (D) Murine CSF1 levels were detected in sera of APAP-ALI mice treated with hCSF1-mFc (1.0 mg/kg, s.c.) or vehicle alone. n.s., not statistically signficant. Each circle represents an individual mouse (B-D).

Supporting Fig. S5. Iterative hCSF1-treatment shows a reduced trend in TUNEL staining in APAP-ALI mice. (A) Scatter plot shows the percentage of TUNEL-positive nuclei in liver sections of APAP-ALI mice treated with or without hCSF1-mFC (1.0 mg/kg, s.c.). Each circle represents an individual mouse. (B) Representative micrographs of TUNEL-stained IF liver sections from APAP-ALI mice treated with hCSF1-mFc (1.0 mg/kg, s.c.) or vehicle alone. Images show DAPI (all nuclei; left column), TUNEL-stained nuclei (568 nm; middle column), and merged images (right column). White arrowheads indicate TUNEL-positive nuclei. Positive control (DNase-treated slides) and negative control (no TdT enzyme applied) slides are provided as indicated. Statistical *P* values are provided. Abbreviation: ALI, acute liver injury; APAP, acetaminophen; DAPI, 4′,6-diamidino-2-phenylindole; TdT, terminal deoxynucleotide transferase; TUNEL, Terminal deoxynucleotidyl transferase dUTP nick end labeling.

Supporting Fig. S6. Gating strategy for *in vivo* phagocytosis assays. Top panels, in sequence (left to right), show all cells used for analysis, exclusion of doublets, and cell viability selection (live/dead stain). CD45-positive cells were selected for against lineage (lin) dump channel. Lineage: Ly6G- CD3- CD19- NK1.1- cells to exclude neutrophils, T cells, B cells, and NK cells respectively. Infiltrating (F4/80^int^, CD11b^hi^) and resident (F4/80^hi^, CD11b^int^) macrophages were identified in the CD45-positive gate. Resident and infiltrating macrophages were further classified based on their Ly6C status (i.e. Ly6Chi or Ly6Clo), and PKH-positivity (i.e. uptake of PKH26-PCL, a soluble fluorescent dye that labels cells performing phagocytosis). Negative PKH gate was set using gating parameters from a healthy mouse liver digest injected with vehicle instead of PKH. Ly6C gating set using fluorescence minus one (FMO) controls. Abbreviations: SSC-A, side-scatter area; FSC-A, forward-scatter area; PKH, Paul Karl Horen (26-phagocytic cell linker dye).

Supporting Fig. S7. Flow cytometry of hepatic macrophage populations from fibrotic mice after iterative hCSF1-mFc reveals a major increase in infiltrating macrophages and phagocytosis. (A) Scatter plots show percentage of cells (of parent population; left), corresponding mean fluorescence intensity values (MFI; centre), and representative PKH uptake histograms (right panel) for infiltrating (top panels) and resident macrophages (bottom panels). (B) Flow cytometry scatter plots of Ly6C status (left), and corresponding histograms (right) in infiltrating and resident macrophages after treatment with hCSF1-mFC or vehicle alone. * *P* < 0.05, ** *P* < 0.01, n.s. not statistically significant. Abbreviations: MFI, mean fluorescence intensity; PKH, Paul Karl Horen (26-phagocytic cell linker dye).

Supporting Fig. S8. *E. Coli*-coated pHrodo® bioparticles are engulfed by hepatic macrophages following intravenous injection and induce a pro-inflammatory response in healthy mice. (A) Individual serum ALT activities (left) and serum albumin levels in healthy mice treated with pHrodo® bioparticles (400 µg, i.v.) or vehicle alone (PBS). (B) Weight loss quantified in individual mice eight hours following treatment with PBS or pHrodo® bioparticles. (C) Neutrophils were detected by Ly6G IHC staining in fixed liver sections. Quantification was performed by image analysis and expressed as average count per 20x field. (D) Representative IHC photomicrographs show Ly6G staining in liver from healthy mice treated with vehicle alone (PBS, left) and *E. Coli* pHrodo® bioparticles (right). (E) Representative IF sections from healthy mice treated with vehicle alone (top row) or *E. Coli* pHrodo® bioparticles (bottom row). (F) Higher magnification of pHrodo sections shown in (E). Images show, in columns, DAPI nuclear stain (cyan, left), F4/80 macrophage stain (yellow, center-left), pHrodo bioparticles (magenta, centre-right), and merged overlay images (right). White arrowheads indicate pHrodo bioparticles that colocalise with F4/80-positive cells. * P < 0.05; *** P < 0.001, n.s. not statistically significant. Abbreviations: ALT, alanine aminotransferase; DAPI, 4′,6-diamidino-2-phenylindole; PBS, phosphate-buffered saline.

Supporting Fig. S9. (A). Gene expression analysis from preparations extracted from whole liver harvested from *E. Coli* / CCl_4_-induced fibrotic mice receiving iterative treatment with either vehicle alone or hCSF1-mFC (1 mg/kg, s.c.). (B) Plasma TNFα * P < 0.05; ** P < 0.01; P < 0.001. Each circle represents an individual animal.

Supporting Fig. S10. Iterative hCSF1-mFc treatment does not increase P21 in hepatocytes in CCl_4_-induced fibrotic mice. (A) Scatter plots shows quantification of P21 (percentage P21-positive nuclei) in treatment groups in fibrotic mice treated with hCSF1-mFC (0.1 – 1.0 mg/kg, s.c.) or vehicle alone. Left panel shows total P21-positive cells. Right panel shows P21 positivity in HNF4α-positive cells only. (B) Plasma TGFβ concentrations in fibrotic mice treated wth hCSF1-mFc or vehicle alone. (C) Representative IF photomicrographs of liver tissue harvested from fibrotic mice treated with hCSF1-mFc or vehicle alone. Panels show fluorescent channels for DAPI (cyan, nuclear marker, left) HNF4α (yellow, hepatocyte-marker, centre-left), P21 (magenta, senescence marker, centre-right), and overlay merged channel (right). Panels shown in treatment groups (0 – 1.0 mg/kg, in rows). Arrowheads indicate nuclei dual positive for HNF4α and P21. n.s., not statistically significant. Abbreviations: CCl_4_, carbon tetrachloride; DAPI, 4′,6-diamidino-2-phenylindole; HNF4α, hepatocyte nuclear factor alpha; TGFβ, transforming growth factor beta.

Supporting Fig. S11. Iterative hCSF1-mFc treatment induces P21 in hepatocytes in healthy and APAP-ALI mice. (A) Scatter plots shows P21 quantification (percentage P21-positive nuclei) in treatment groups in healthy mice treated with hCSF1-mFC (0.1 – 1.0 mg/kg, s.c.) or vehicle alone. Left panel shows total P21 positivity in all nuclei. Right panel shows P21 positivity in HNF4α-positive cells only. (B) Scatter plots show P21-positivity in APAP-ALI mice treated with vehicle alone or hCSF1-mFc (1.0 mg/kg, s.c.). Total P21-positivity (left) and hepatocyte P21-positivity (right) are shown in treatment groups. Circles represent individual mice. (C) Representative IF photomicrographs of liver tissue harvested from healthy mice treated with hCSF1-mFc or vehicle alone. Panels show fluorescent channels for DAPI (cyan, nuclear marker, left) HNF4α (yellow, hepatocyte-marker, centre-left), P21 (magenta, senescence marker, centre-right), and overlay merged channel (right). Panels shown in treatment groups (0 – 1.0 mg/kg, in rows). (D). Representative HNF4α and P21 staining in liver tissue harvested from APAP-ALI mice treated with vehicle alone (top row) or hCSF1-mFc (bottom row). Arrowheads indicate nuclei dual positive for HNF4α and P21. * P < 0.05; ** P < 0.01. Abbreviations: APAP-ALI, acetaminophen-induced acute liver injury; DAPI, 4′,6-diamidino-2-phenylindole; HNF4α, hepatocyte nuclear factor alpha.

| **Antigen** | **Supplier** | **CAT#** | **Host** | **Stock conc. (mg/mL)** | **Dilution** | **Antigen retrieval** |
| --- | --- | --- | --- | --- | --- | --- |
| F4/80 (IHC) | Abcam | ab6640 | Rat | 1.0 | 1:50 | Proteinase K (125 µg/mL, 5 minutes, 37 °C) |
| BrdU (IHC, after F4/80) | Abcam | ab6326 | Rat | 1.0 | 1:100 | Na Citrate (pH 6.0) buffer, 15 minutes microwave heating |
| Ly6G (IHC) | Biolegend | 127602 | Rat | 0.50 | 1:1000 | 10 minutes, sodium citrate (pH 6.0) |
| HNF4α (IF) | Perseus proteomics | PP-1415 | Mouse | 0.1 | 1:200 | Tris-EDTA + Tween 20 (pH 9.0), 15 minutes microwave heating |
| BrdU (IF) | Abcam | ab6326 | Rat | 1.0 | 1:200 | Tris-EDTA + Tween 20 (pH 9.0), 15 minutes microwave heating |
| P21 (IF) | Abcam | Ab188224 | Rabbit | 0.487 | 1:200 | Tris-EDTA + Tween 20 (pH 9.0), 15 minutes microwave heating |
| CSF1R (cryo/IF) | Abcam | ab183316 | Rabbit | 0.096 | 1:50 | None |
| F4/80 (cryo/IF) | Abcam | ab6640 | Rat | 1.0 | 1:50 | None |

**Supporting Tables**

**Table S1:** Antibody details for immunodetection assays

| **Gene Name** | **Quantitect Assay ID** |
| --- | --- |
| *Mmp8* | QT00113540 |
| *Mmp9* | QT00108815 |
| *Mmp13* | QT00111104 |
| *Acta2* | QT00140119 |
| *Arg1* | QT00134288 |
| *Mrc1* | QT00103012 |
| *Nos2* | QT00100275 |
| *Ccl2* | QT00167832 |

**Table S2:** Primer assays used for gene expression analysis

**Supporting Materials and Methods**

***Human monocyte isolation and culture*.** Peripheral blood mononuclear cells (PBMCs) were isolated from healthy donor buffy coats (Swiss Red Cross, Bern, Switzerland; approved by the Ethikkommission Nordwest-und Zentralschweiz) using the SEPMATE-50 (#15460, StemCell Technologies). Briefly, 15 ml of Ficoll-density gradient was added to the SepMate tubes (#1114547, Lymphoprep, Axis-Shield) after which 20 ml of diluted blood was added. Samples were centrifuged at 300 g for 8 min at room temperature. The top layer containing the PBMCs was collected and washed twice with PBS containing 2 % human serum (#HP1022, Valley Biomedical). Red cell lysis buffer (#79217, Qiagen) was added and left on ice for 4 min before PBS - 2% human serum was added. Monocytes were then isolated by magnetic sorting using a human CD14 negative selection kit (#19059 Easysep, Stemcell Technologies) according to the manufacturer’s instructions. Monocytes were frozen at -80 °C in 60 % HBS buffer (NaCl 130 mM, NaH_2_PO_4_ 0.9 mM, MgSO_4_ 0.8 mM, KCl 5.4 mM, glucose 25 mM, HEPES 20 mM, pH7.4), 30 % human serum, 10 % DMSO until use. Monocyte purity was assessed by CD14 flow cytometry (CD14 #557742 and IgG2a isotype control #557907, BD Biosciences). After 30 min of antibody stain on ice, the cells were washed with PBS containing 2 % human serum and 1 mM EDTA, fixed with 1 % PFA (Electron Microscopy Sciences) in PBS, washed and read with a flow cytometer (CyFlow Space, Partec; CyAn ADM, Beckman Coulter; BD FACSCanto II, BD Biosciences). The monocyte purity was 80 – 95 %.

***Rezasurin assay.*** Monocytes were plated at 7500 cells / well in 384-well plates (Greiner, black, #78194) in 12.5 µL IMDM medium or RPMI medium (#52400-025 Invitrogen, Thermo Fisher Scientific) supplemented with 2 % fetal calf serum (FCS), 1 % Glutamax (#35050038 Invitrogen, Thermo Fisher Scientific) and 1 % Penicillin/Streptomycin. For the treatments, 12.5 µL of 2x concentrated CSF1 or test constructs in culture medium were added and the cells were incubated for 3 days at at 37 °C, 5 % CO_2_. Resazurin assay (#11884, Cell signaling) was performed after 4 hours incubation with the reagents, according to the manufacturer’s instructions. Data were analysed as fluorescence signal measured at 560 and 590 nm on an Envision plate reader (PerkinElmer).

***ERK phosphorylation assay.*** Monocytes were thawed and plated in 96-well plates at 9x10^4^ cells / well in 100 µL IMDM (#21056023 Invitrogen, Thermo Fisher Scientific) supplemented with 0.5 % human serum, 1 x NEAA (#11140035 Invitrogen, Thermo Fisher Scientific), 2 mM Glutamax (#35050038 Invitrogen, Thermo Fisher Scientific), 1 mM Na-pyruvate (#11360039 Invitrogen, Thermo Fisher Scientific), 4 µg/mL human insulin (#12585014 Invitrogen, Thermo Fisher Scientific), 5 ng/mL human recombinant CSF1 (#216MC-025, R&D Systems) and 1 % Penicillin/Streptomycin (#4-01F00-H, BioConcept). After 4 hours at 37°C, 5 % CO2, medium was changed for overnight starvation in IMDM medium without CSF1. The next day, medium was removed and CSF1 or equivalent molar concentrations of test constructs, diluted in medium without serum, were added for 5 min. Cells were then lysed and Erk phosphorylation was monitored in 384-well plates (white, Greiner) according to the manufacturer’s instructions (#64ERKPEH, Cisbio). Data were analysed as the ratio of 655/620nm values.

***Immunostaining assays.*** Liver tissue was perfused (PBS) and formalin-fixed with other organs (12-16 hours) before paraffin embedding. Four micron sections were cut from formalin-fixed paraffin-embedded (FFPE) blocks and collected on Superfrost Plus™ slides (Thermo Fisher). Sections were dewaxed and hydrated, before heat-induced antigen retrieval using 800 W microwave heating. Sections were washed in PBS before blocking (30-60 minutes) in protein solution (Spring Bioscience, Pleasanton, CA). Primary antibodies specific to proteins of interest (or isotype antibody controls) were incubated overnight (4 °C). For IF, fluorophore-conjugated secondary antibodies were incubated (1:200, 1 hour) before and after PBS washing, and then counterstained with DAPI (1 μg/mL, 15 minutes) to visualise nuclei. IF sections were mounted (Fluoromount-G, Thermo Fisher) and imaged on an Operetta high content imaging system (PerkinElmer) and quantified in Columbus™ software (PerkinElmer). For IHC stains, additional blocking was performed using Bloxall (Vector labs) and Avidin/biotin block (Invitrogen, [Thermo Fisher]). Biotinylated secondary antibodies were incubated (1:500, 1 hour) before avadin-based peroxidase treatment. IHC stains were developed by application of DAB chromogen (2-5 minutes, room temperature; Dako, Glostrup, Denmark). For dual IHC stains, some antigens were visualised by developing Vector® red Alkaline phosphatase substrate (2 hours, room temperature, Vector Labs) after avidin-based alkaline phosphatase (Vector Labs) treatment. Vector® red is heat stable and was performed first, before subsequent heat-induced antigen retrieval steps. Slides were counterstained (haematoxylin), dehydrated, and mounted in Pertex. IHC stains were imaged using a Vectra® Polaris™ imaging system (PerkinElmer), and quantified using inForm® software (PerkinElmer). A minimum of 8 non-overlapping fields were quantified, per sample. Antigen retrieval and antibody incubation conditions are antibody-specific and are summarised in Table 1.

***Cryostaining (IF).*** Liver tissue was perfused (PBS) and immediately frozen on dry ice after tissue harvest. Seven micron cryosections were cryosections from frozen liver tissue (-18 °C) on a cryostat and collected on Superfrost Plus™ slides (Thermo Fisher). Cryosections were post-fixed in ice-cold methanol:acetone (1:1; 5 minutes) before air-drying (20 minutes, room temperature). Cryosections were hydrated with PBS (5 minutes), protein blocked (30 minutes), and incubated with primary antibody overnight. Antigens were visualised using IF before scanning and analysis steps, as detailed above.

***TUNEL staining.*** TUNEL staining was performed using the *in situ* cell death detection kit (Roche, Basel, Switzerland) TMR red as per manufacturer’s instructions. Briefly, FFPE sections were dewaxed and hydrated. Antigen retrieval was performed by proteinase K treatment (20 μg/mL, 20 minutes, room temperature). A positive control section was generated by incubating a tissue section with DNase I (10 U/mL, 10 minutes, room temperature). Sections were treated with a labelling solution containing (TMR-labelled nucleotides with Terminal deoxynucleotidyl transferase) before extensive washing, DAPI counterstaining, and mounting (as above). A negative control slide was treated with labelling solution without enzyme. TUNEL-slides were imaged on Operetta Operetta high content imaging system (PerkinElmer) and quantified in Columbus™ software (PerkinElmer).

***Histology.*** Four micron FFPE sections were stained with haematoxylin and eosin (H+E) using a Shandon Varistain Gemini ES Automated Slide Stainer (ThermoScientific) and mounted using the Shandon ClearVue Coverslipper (ThermoScientific). Histology images were captured on a Vectra® Polaris™ imaging system (PerkinElmer). Picrosirius Red staining (PSR) was performed essentially as described previously [1].

***RT-PCR****.* For tissue, total RNA was extracted from whole liver tissue by phenol extraction. Briefly, whole liver tissue (20 mg) was homogenised in 1 mL Trizol (Invitrogen) using TissueRuptor. Homogenates were shaken vigorously and left to incubate for 5 minutes (room temperature). Chloroform (200 µL) was added to each tube and shaken vigorously (15 seconds). Preparations were centrifuged (12,000 g, 15 minutes, 4 °C) and the upper aqueous phase was separated (350 µL) and mixed with isopropanol (500 µL) to precipitate RNA. RNA was pelleted by centrifugation and washed in fresh 75 % ethanol. Washed RNA was centrifuged and supernatant discarded. RNA pellet was allowed to air-dry for 5 minutes before dissolution in 100 µL RNase-free water (Qiagen, Hilden, Germany). RNA was quantified by Nanodrop ND-1000 (Thermo Fisher) and reverse transcribed using Quantitect® reverse transcription kit (Qiagen), with genomic DNA removal step included (as per manufacturer’s instructions). Gene expression was quantified using QuantiTect SYBR Green PCR Kit (Qiagen) with Quantitect primers (see Table 2) and assayed in duplicate on a 384-well format on a Roche Lightcycler 480 II (Roche). Relative gene expression was quantified using 2*^-^*^ΔΔCt^ method using *Gapdh* as the housekeeping gene. For gene expression of cultured BMDMs, RNA was harvested using RNeasy mini kit (Qiagen) instead, and *18S rRNA* served as the housekeeping gene.

***Liver digest, leukocyte digestion, and flow cytometry.*** The left lateral lobe was enzymatically digested and non-parenchymal cells (NPCs) isolated using the protocol essentially as described previously [2]. 1x10^6^ NPCs were labelled with an antibody cocktail consisting of CD11b BV650 (clone M1/70; eBioscience), Ly-6C V450 (clone HK1.4; eBioscience), CD45.2 AF700 (clone 104; eBioscience), F4/80 APC (1:100, clone BM8; Invitrogen), Ly-6G PE-Cy7 (clone 1A8; BioLegend), CD3 PE-Cy7 (clone 17A2; BioLegend), NK1.1 PE-Cy7 (clone PK136; BioLegend), CD19 PE-Cy7 (clone 6D5; BioLegend), CD31 PE-Cy7 (clone 390, eBioscience). Cell viability was assessed with Fixable Viability Dye eFluor780 (1:1000, eBioscience) according to manufacturer’s protocols. Flow cytometry was performed on an LSRII Fortessa (BD Biosciences, Franklin Lakes, NJ) flow cytometer. Analysis was performed in FlowJo10 software (Tree Star Inc, Ashland, OR). Gating strategy is provided in Supporting Fig. S6. Gates were set using unstained and fluorescence minus one (FMO) thresholds.

***Hematology.*** Whole blood was collected in EDTA-blood tubes and stored at room temperature. Hematology (red blood cells, white blood cells, platelets) was performed immediately on a Celltac α analyzer (Nihon Kohden, Tokyo, Japan) following manufacturer’s instructions.

**Supporting References**

[1]	Esser H, Kilpatrick AM, Man TY, Aird R, Rodrigo-Torres D, Buch ML, et al. Primary cilia as a targetable node between biliary injury, senescence and regeneration in liver transplantation. J Hepatol 2024. https://doi.org/https://doi.org/10.1016/j.jhep.2024.06.002.

[2]	Lewis PS, Campana L, Aleksieva N, Cartwright JA, Mackinnon A, O’Duibhir E, et al. Alternatively activated macrophages promote resolution of necrosis following acute liver injury. J Hepatol 2020;73:1–12. https://doi.org/10.1016/j.jhep.2020.02.031.
